# A Cross-Sectional Study of the Cardiovascular Effects of Welding Fumes

**DOI:** 10.1371/journal.pone.0131648

**Published:** 2015-07-06

**Authors:** Huiqi Li, Maria Hedmer, Monica Kåredal, Jonas Björk, Leo Stockfelt, Håkan Tinnerberg, Maria Albin, Karin Broberg

**Affiliations:** 1 Division of Occupational and Environmental Medicine, Laboratory Medicine, Lund University, Lund, Sweden; 2 Competence Centre for Clinical Research, Lund University, Lund, Sweden; 3 Department of Occupational and Environmental Medicine, Sahlgrenska University Hospital and Academy, University of Gothenburg, Gothenburg, Sweden; 4 Unit of Metals & Health, Institute of Environmental Medicine, Karolinska Institutet, Stockholm, Sweden; University of British Columbia, CANADA

## Abstract

**Objectives:**

Occupational exposure to particulate air pollution has been associated with an increased risk of cardiovascular disease. However, the risk to welders working today remains unclear. We aimed to elucidate the cardiovascular effects of exposure to welding fumes.

**Methods:**

In a cross-sectional study, structured interviews and biological sampling were conducted for 101 welders and 127 controls (all non-smoking males) from southern Sweden. Personal breathing zone sampling of respirable dust was performed. Blood pressure (BP) and endothelial function (using peripheral arterial tonometry) were measured. Plasma and serum samples were collected from peripheral blood for measurement of C-reactive protein, low-density lipoprotein, homocysteine, serum amyloid A, and cytokines.

**Results:**

Welders were exposed to 10-fold higher levels of particles than controls. Welders had significantly higher BP compared to controls, an average of 5 mm Hg higher systolic and diastolic BP (P≤0.001). IL-8 was 3.4 ng/L higher in welders (P=0.010). Years working as a welder were significantly associated with increased BP (β=0.35, 95%CI 0.13 – 0.58, P=0.0024 for systolic BP; β=0.32, 95%CI 0.16 – 0.48, P<0.001 for diastolic BP, adjusted for BMI) but exposure to respirable dust was not associated with BP. No clear associations occurred between welding and endothelial function, or other effect markers.

**Conclusions:**

A modest increase in BP was found among welders compared to controls suggesting that low-to-moderate exposure to welding fumes remains a risk factor for cardiovascular disease.

## Introduction

Inhalation of particulate matter (PM) air pollution is associated with an increased risk of cardiovascular disease (CVD), with no clear exposure threshold [1–4] and fine particles (PM with aerodynamic diameter < 2.5 μm) play a particularly important role [5]. Occupational air pollution, which may cause chronic and high-level exposure to PM, was associated with an increased risk of CVD [6, 7]. Therefore, PM exposure in occupational settings may affect workers’ health.

Welders have high levels of exposure to fine particles that consist mainly of metal oxides, as most welding uses metal alloys containing iron, manganese, chromium, and nickel. Usually welding fumes have much higher particle concentrations than outdoor ambient air [8–10]. Previous reports suggested an association between welding fumes and increased risk of CVD [6, 11, 12]; for example, the standardized incidence ratio for acute myocardial infarction was 1.12 (95% CI 1.01–1.24) in a Danish prospective study of welders followed until 2006 [12] and the standardized mortality ratio for ischemic heart disease was 1.35 (95% CI 1.1–1.6) in a Swedish study of welders followed until 1995 [6]. However, one meta-analysis revealed that the relationship between welding fumes and CVD was not robust, since bias and confounding effects could not be ruled out [13]. Therefore, the association between occupational exposure to welding fumes and elevated CVD risk was not well established, despite of the general consensus that exposure to PM increases the risk for CVD. Also, the studies on Swedish and Danish welders were carried out more than 10 years ago, and although there are no indications that the exposure levels have decreased since then, the precautionary measures used by workers might have improved, including general mechanical ventilation systems, local exhaust ventilation, welding guns with integrated exhaust ventilation, and personal protective equipment such as powered air purifying respirators (PAPRs). Thus, it is not known if the current working conditions of welders in Sweden continue to pose a risk for cardiovascular toxicity.

Despite the observational data, the mechanisms coupling exposure to fine particles with adverse cardiovascular events need further clarification. Different studies have reported the intermediate steps of CVD induced by PM, such as systemic oxidative stress and inflammation [14–17], endothelial dysfunction [18–21], and decreased heart rate variability [22, 23]. Exposure to fine particles has been associated with increased levels of interleukin 6 (IL-6), C-reactive protein (CRP), serum amyloid A (SAA) [24–26], and traffic-related particles with increased levels of homocysteine [27]. These markers appear to be predictive for CVD [28–31], probably because IL-6, CRP, and SAA reflect inflammation and hyperhomocysteinemia reflects accelerated atherosclerosis and activation of proinflammatory factors [32]. PM exposure could damage endothelial function, and PM exposure has been shown to be an independent predictor of CVD and mortality [33, 34]. Endothelial function has so far usually been measured in the clinic, due to the lack of non-invasive equipment that can easily be used in a field setting. Here, we measured endothelial function using Endo-PAT, a non-invasive method that assesses endothelial function via peripheral arterial tonometry with a portable device [35]. Furthermore, increased blood pressure (BP) is a well-known risk factor for cardiac disease and for stroke, so it is important to know if BP is part of the causal pathway.

The aim of our study was to evaluate if long-term exposure to welding fumes results in adverse effects on the cardiovascular system in welders.

## Materials and Methods

### Study participants

We recruited 101 welders and 127 controls in this study. The welders were from 10 medium-sized companies in southern Sweden, producing heavy vehicles, lifting tables, stoves, heating boilers and pumps, and equipment for the mining industry. All 10 welding companies used gas metal arc welding with mild steel (low-carbon steel, containing no chromium or nickel). Characteristics for 8 of the companies have recently been described [36], and the compositions of the welding fumes in the companies were found to be relatively homogenous. The controls were from 7 companies in southern Sweden, of which 6 companies were storage houses organizing goods and one company was housing company working as gardeners. All controls had no known occupational exposure to welding fumes or particles. All participants were male workers that were currently non-smoking.

The enrolment was carried out in 2010 and 2011. The workers went through a structured interview about ethnicity (participants’ and their parents’ nationality, categorized as European or not), education, personal disease history, family CVD and cancer history, prescription and non-prescription medication, frequency of vegetable intake, frequency of fruit intake, frequency of fish intake, physical activity, smoking history, passive smoking, use of snus (a moist powdered tobacco product consumed in Sweden), alcohol consumption, current residence, wood burning stove/boiler at home, wood smoke from the neighbourhood, exposure to traffic, work task, protection device, occupational history, and hobbies with exposure to particles (i.e. welding fumes or diesel exhaust).

On the same day as the structured interview, measurements of BP, endothelial function, and sampling of blood were performed. The blood sampling and blood pressure measurement were performed in the early half of the work shift, and endothelial function was measured in the latter half. The interview, blood pressure, blood sampling, and endothelial function were all performed in rooms separate from the working area; these rooms were generally without PM, or noise. Exposure measurement (i.e. measurement of respirable dust) was performed after the day of interview with a median time difference of 2 months (range 0–9 months).

### Ethics Statement

All study participants gave informed written consent to take part in the study and the study was approved by the Regional Ethical Committee of Lund University.

### Exposure assessment

The concentration of welding fumes was measured as respirable dust, since the particles in welding fumes are typically around 0.5 μm in size [9, 36]. An occupational hygienist collected the air samples in the workers’ breathing zone on pre-weighed 37 mm mixed cellulose ester filters (0.8 μm pore size) fitted in cassettes attached to Respirable Dust Cyclones (BGI4L, BGI Inc., USA; 50% cutoff at an aerodynamic equivalent particle diameter of 4 μm). The airflow was set at 2.2 L/min and regularly checked before, during, and after sampling. The sampling time was on average 6.9 h. The filters were weighed to measure the collected amounts of respirable dust. Personal breathing zone sampling of respirable dust was performed for 53 welders in the study plus 17 welders who had a similar exposure but did not donate blood samples or participate further in the study. If the welders used PAPRs, the air outside the respirators was sampled. Personal breathing zone samples were also collected in 2 control companies for 19 workers. Stationary measurement was conducted in another 4 control companies with a direct-reading instrument (Sidepak Model AM510, TSI Inc., MN USA).

PAPRs were used by 49% of the welders that participated in the exposure measurements. Parallel samplings inside and outside of PAPRs showed that the respirable dust concentrations were at least 3 times lower inside the PAPRs. Based on this result and published data on workplace protection factors [36–39], the concentrations of respirable dust measured outside the PAPRs were reduced by a factor of 3 to estimate the exposure inside the PAPRs. The exposure of participants with no measurement of respirable dust was assessed by exposure data from workers at the same companies with measurements and with similar tasks.

### Blood pressure and endothelial function

BP was measured in a lying position, using a mercury sphygmomanometer for each participant after the structured interview (interview time around 15 min).

Analysis of endothelial function was performed using the Endo-PAT2000 (Itamar Medical Ltd, Caesarea, Israel) according to the manufacturer’s recommendations. At least 4 hours passed between measurement of BP and analysis with Endo-PAT, and the participants rested in a lying position for 15 min before the test. The analysis was performed in quiet and, if possible, low-light circumstances at 20–22°C, and a blanket was offered if the participant felt cold. Pletysmographic finger probes measuring digital pulse wave amplitudes were placed on the index fingers of both hands. The right arm was tested, while the left arm served as the control. The examination consisted of 5 min baseline recording, 5 min of ischemia caused by right brachial artery occlusion using the sphygmomanometer cuff, and 5 min recording of the post-occlusion reactive hyperemia. The cuff pressure was generally set at 220 mm Hg, unless pulse signals were detected, in which case higher pressure was applied (not more than 250 mm Hg). Data was stored digitally. Reactive hyperemia index (RHI), augmentation index (AI@75), and heart rate were automatically calculated in a user-independent manner by the Endo-PAT software (version 3.3.2; Itamar Medical). RHI and AI@75 are computed indexes and therefore arbitrary values without units. Higher RHI values reflect better endothelia function and lower AI@75 values (including negative results) reflect better arterial elasticity. The algorithm for RHI compares the pulse wave amplitudes after ischemia with the baseline amplitudes while adjusting for changes in the control finger, and the algorithm for AI@75 compares the systolic peak and reflected wave’s peak and further normalized to a heart rate of 75 bpm.

### Markers for inflammation and risk of CVD

Venous blood was obtained from each participant. Heparin plasma, EDTA plasma, and serum were isolated in the field 10 min after sampling and transported to the laboratory on dry ice. Plasma and serum samples were stored at -80°C until analysis.

In heparin plasma, the following markers were measured: CRP by immunoturbidimetry, low-density lipoprotein (LDL) by selective micellar solubilization, and homocysteine by an indirect enzymatic method measuring the absorbance of NAD^+^. In serum, SAA was analyzed by immunonephelometry. All measurements were performed at the Department of Clinical Chemistry in Lund University Hospital, and used standard protocols.

In EDTA plasma, cytokines related to CVD or inflammation [IL-1β, IL-6, IL-8, granulocyte colony-stimulating factor (G-CSF), monocyte chemotactic protein-1 (MCP-1), macrophage inflammatory protein-1β (MIP-1β), tumor necrosis factor α (TNF-α), and vascular endothelial growth factor (VEGF)] were measured by the use of Luminex XMAP technology on a Bio-plex 200 platform (Bio-Rad, Hercules, CA, USA), according to the instructions from the manufacturer. The results were evaluated in Bio-Plex manager 6.0 (Bio-Rad). The standard points were fitted by a 5 parameter logistic model to the standard curve and the fit probabilities were in the range of 0.44–0.78. The between-day precision for a control serum sample was determined as the coefficient of variance: IL-8 (19%), G-CSF (16%), MCP-1 (13%), MIP-1β (10%), TNF-α (53%), VEGF (26%).

### Statistical analysis

The characteristics and concentrations of markers for the welders and controls were compared by Mann-Whitney U tests. The percentages of personal/family history of CVD and medication for CVD were compared by Fisher’s exact tests. Some participants had missing values for some variables, but they were included in the analysis when possible. The distributions of SAA and MIP-1β were skewed and their values were natural log-transformed.

The general linear model was adopted to examine differences of outcomes (BP, CRP, SAA, homocysteine, RHI, AI@75, and cytokines) between welders and controls. The associations between outcomes and years working as a welder and exposure to respirable dust were investigated by general linear model in welders only to evaluate the dose-response of exposure to welding fumes, both long-term and short-term. In the partly adjusted models, age and BMI were included as continuous variables. Besides age and BMI, the fully adjusted models included possible confounders and the criterion of inclusion was bivariate correlations (Pearson correlation) with both systolic and diastolic BP with P < 0.20. In the fully adjusted model, further adjustments were made for ethnicity (2 categories, European versus non-European), education (2 categories, high school or lower versus university or higher), physical activity (4 ordinal categories, from sedentary type to highly physically active), family history of CVD (2 categories, yes versus no), smoking history (2 categories, yes versus no), and current residence (2 categories, big city versus non big city) in the analysis of both welders and controls, and for family history of CVD and current residence, in the analysis of welders only. However, when investigating the effect of working years as a welder, models with and without age adjustment were both performed, since age and working years as a welder were highly correlated (Spearman’s correlation rs = 0.75).

All statistical analyses were completed by using SPSS 21.0 (SPSS Inc, Chicago, IL, USA) and statistical significance refers to P<0.05 (two-tailed).

## Results

All outcomes were available for most of the subjects. There were only 2 welders with missing values for respirable dust, 1 control with missing CRP, and 1 control with missing smoking history. The welders and controls showed similar median age and BMI (Table 1). The welders had been working in the current companies for 7 years (range 0–31 years) on average, and the controls for 6 years (range 0–40 years). When taking working experience with welding from different companies into consideration, the welders had been working with welding for 15 years on average, with only 2 welders who had worked for less than 1 year. The welders were exposed to welding fumes measured as respirable dust, with a median concentration of 1.1 mg/m^3^ (geometric mean 1.2 mg/m^3^), whereas the exposure level among the controls was lower than 0.1 mg/m^3^ (P<0.001). The median systolic BP values in both groups were in the “high normal” (systolic BP 120–139 mm Hg or diastolic BP 80–89 mm Hg) range according to the ESH-ESC Guidelines [40]. The welders had significantly higher systolic and diastolic BP compared to controls (P≤0.001). Self-reported CVD was similar in welders and controls. However, twice as many welders reported medication related to CVD and slightly more welders reported a family history of CVD, but these differences were not significant (Table 1). The most common CVD reported was hypertension in both groups and most of the medication for CVD was related to hypertension (Table 2). CRP, LDL, homocysteine, and SAA did not significantly differ between welders and controls (P>0.13, Table 1). Endothelial function (measured as RHI) was not significantly different between welders and controls. For four out of the eight cytokines measured, more than half of the samples were below the limit of detection: 98% of IL-1β, 88% of TNF-α, 83% of IL-6, 56% of G-CSF, 26% of VEGF, 6.9% of IL-8, 1.1% of MCP-1, 0.4% of MIP-1β were below the limit of detection. Therefore, IL-1β, TNF-α, IL-6, and G-CSF were not included in further analysis. Only IL-8 showed significantly higher levels in welders than controls (P = 0.022).

**Table 1:** Basic characteristics and biomarkers in welders and controls.^a^

	Welders N = 101			Controls N = 127			
	Median	5–95%	Count	Median	5–95%	Count	P^e^
Age	41	23–60		43	23–56		0.90
BMI (kg/m^2^)	28	22–34		27	22–34		0.48
Respirable dust (mg/m^3^)	1.1	0.2–8.4		0.1	0.1–0.1		<0.001
Years working as a welder (year)	15	1–38			—		—
Systolic blood pressure (mm Hg)	130	115–155		125	105–145		<0.001
Diastolic blood pressure (mm Hg)	75	60–85		70	60–85		0.001
C-reactive protein (CRP; mg/L)	1.2	0.30–5.0		1.1	0.30–5.0		0.13
Serum amyloid A (SAA; mg/L)	2.3	0.95–8.7		2.4	0.40–14		0.61
Low-density lipoprotein (LDL; mmol/L)	3.1	1.9–4.7		3.1	1.9–4.6		0.49
Homocysteine (μmol/L)	11	8.0–19		12	7.0–16		0.88
Reactive hyperemia index (RHI)	1.8	1.2–3.1		1.8	1.3–2.9		0.90
Augmentation Index (AI@75)	-8.7	-30–14		-10	-29–15		0.76
IL-8 (ng/L)	8.2	3.0–17		6.2	<LOD–12		0.0027
MCP-1 (ng/L)	34	11–88		34	8.4–67		0.32
MIP-1β (ng/L)	129	73–279		119	56–225		0.18
VEGF (ng/L)	43	<LOD–167		31	<LOD–157		0.26
Smoking history (yes/no)			43/58			43/83	0.21^f^
Personal history of CVD (yes/no)^b^			23/78			25/102	0.63^f^
Current use of medication for CVD (yes/no)^c^			11/90			7/120	0.15f
Family history of CVD (yes/no)^d^			44/57			46/81	0.28f

^a^ LOD = limit of detection. CVD = cardiovascular disease.

^b^ Participants reported if they have had myocardial infarction, angina pectoris, hypertension, stroke, blood clot, and/or other CVD.

^c^ Participants reported taking prescription medication for CVD.

^d^ Participants reported if their parents or siblings had had myocardial infarction, stroke, and/or hypertension before they were 65 years old.

^e^ The P-values were derived from Mann-Whitney U tests unless marked with “f” meaning the P-value was from Fisher’s exact test.

**Table 2:** Self-reported CVD, medication for CVD, and family history of CVD in welders and controls.

	N (%)	
	Welders	Controls
Self-reported CVD		
Myocardial infarction	0 (0%)	0 (0%)
Angina pectoris	6 (5.9%)	6 (4.7%)
Hypertension	19 (19%)	16 (13%)
Stroke	0 (0%)	1 (0.79%)
Blood clot	0 (0%)	2 (1.6%)
Other heart disease	1 (1.0%)	2 (1.6%)
Sum^a^	23 (23%)	25 (20%)
Medication for CVD		
Beta blocker	2 (2.0%)	2 (1.6%)
Angiotensin II inhibitor	3 (3.0%)	1 (0.79%)
ACE inhibitor	3 (3.0%)	1 (0.79%)
Diuretic	0 (0%)	3 (2.4%)
Cadmium channel blocker	0 (0%)	1 (0.79%)
Statin	2 (2.0%)	2 (1.6%)
Cortisone	1 (1.0%)	2 (1.6%)
Serotonin receptor inhibitor	1 (1.0%)	0 (0%)
Unspecified medication for hypertension	1 (1.0%)	1 (0.79%)
Sum^a^	11 (11%)	7 (5.5%)
Family history of CVD		
Myocardial infarction	11 (11%)	20 (16%)
Stroke	8 (8.0%)	12 (9.4%)
Hypertension	38 (38%)	34 (27%)
Sum^a^	44 (44%)	46 (36%)

^a^ The sum number does not reflect simple addition, since some participants fell in more than one category.

In general linear models, the welders showed significantly higher systolic and diastolic BP and IL-8. There were no other significant differences between welders and controls. When taking possible confounders and covariates into consideration, the effect estimations were somewhat reduced, but the level of significance did not change (Table 3). Heart rate did not differ between welders and controls (β = 1.5, P = 0.23). In a sensitivity analysis, we excluded participants with a personal history of CVD; the BP differences between welders and controls were lower for systolic BP and somewhat higher for diastolic BP, but still significant (β = 5.8, P<0.001 for systolic BP; β = 4.0, P = 0.0016 for diastolic BP, fully adjusted). For the cytokines, a sensitivity analysis was performed to adjust for batch variances; however, the results were very similar.

**Table 3:** Differences in blood pressure and biomarkers between welders and controls.

	Unadjusted			Partly adjusted^a^			Fully adjusted^b^		
Outcome	Beta	95% CI	P	Beta	95% CI	P	Beta	95% CI	P
Systolic BP	8.3	5.1–11	<0.001	8.2	5.1–11	<0.001	7.7	4.6–11	<0.001
Diastolic BP	4.1	1.8–6.5	<0.001	4.2	2.0–6.4	<0.001	3.9	1.6–6.2	<0.001
CRP	0.16	-0.45–0.78	0.61	0.14	-0.47–0.75	0.65	0.056	-0.58–0.69	0.86
SAA^c^	0.043	-0.18–0.27	0.70	0.034	-0.19–0.25	0.76	-0.0081	-0.24–0.22	0.95
LDL	-0.10	-0.34–0.14	0.41	-0.11	-0.33–0.11	0.33	-0.081	-0.32–0.15	0.49
Homocysteine	0.43	-1.2–2.0	0.61	0.43	-1.2–2.1	0.60	0.25	-1.4–1.9	0.77
RHI	0.024	-0.13–0.18	0.77	0.025	-0.13–0.18	0.76	0.014	-0.15–0.18	0.87
AI@75	-0.14	-3.6–3.4	0.94	-0.31	-3.1–2.4	0.82	-1.4	-4.2–1.4	0.33
IL-8	3.4	0.77–6.0	0.011	3.4	0.82–6.0	0.010	3.3	0.64–6.0	0.015
MCP-1	4.4	-0.89–9.6	0.10	4.3	-0.93–9.6	0.11	3.6	-1.8–9.0	0.20
MIP-1β^c^	0.064	-0.50–0.18	0.27	0.060	-0.051–0.17	0.29	-0.031	-0.084–0.15	0.59
VEGF	11	-3.7–25	0.14	11	-3.9–25	0.15	8.5	-6.3–23	0.26

Effect estimates presented are β-values for occupation (welder/control) derived from general linear models for each outcome.

^a^ The partly adjusted model was adjusted for age and BMI.

^b^ The fully adjusted model was adjusted for age, BMI, ethnicity, education, activity, family history of CVD, smoking history, and current residence.

^c^ SAA and MIP-1β were natural log-transformed.

Internal analysis was then performed in the group of welders. Working years as a welder was highly correlated with age (Spearman’s correlation rs = 0.75). When both working years as a welder and age were included in the model, the standard error of working years increased by approximately 50% (for instance, in the model where systolic BP was the outcome, standard error for working years was 0.11 in unadjusted model and 0.17 when age was included). This result indicated that the colinearity of years working as a welder and age was hampering the model fit. However, the model with age adjustment could still provide information about the strength of association with the outcome. The number of years working as a welder was positively associated with systolic and diastolic BP, and the associations were still significant after adjustment for age and BMI (Tables 4 and 5), with an average increase of 3.9 mm Hg in systolic BP and 3.5 mm Hg in diastolic BP per 10 years working as a welder. When excluding participants with a personal history of CVD, the association between systolic BP and working years as a welder became non-significant (β = 0.11, P = 0.36, fully adjusted) but association between diastolic BP and working years was still significant (β = 0.20, P = 0.030, fully adjusted). LDL and AI@75 showed significant associations with years working as a welder in the expected direction. However, these significant associations disappeared when age was included (Table 5), indicating that these biomarkers were more influenced by age than by years working as a welder. Self-reported CVD was associated with years working as a welder in the unadjusted model [odds ratio (OR) = 1.1, 95% CI 1.0–1.1, P = 0.0062, logistic regression], but the association was not significant after adjusting for age, BMI, and other covariates (OR = 1.0, 95% CI 0.96–1.1, P = 0.42).

**Table 4:** The effect of years working as a welder on blood pressure and biomarkers in welders.

	Unadjusted			Partly adjusted^a^			Fully adjusted^b^		
Outcome	Beta	95% CI	P	Beta	95% CI	P	Beta	95% CI	P
Systolic BP	0.39	0.17–0.61	<0.001	0.35	0.13–0.58	0.0024	0.31	0.088–0.52	0.0064
Diastolic BP	0.35	0.19–0.51	<0.001	0.32	0.16–0.48	<0.001	0.29	0.13–0.45	<0.001
CRP	-0.0042	-0.045–0.037	0.84	-0.012	-0.053–0.029	0.23	-0.0050	-0.046–0.036	0.81
SAA^c^	0.0036	-0.011–0.018	0.63	0.00052	-0.014–0.015	0.94	0.00049	-0.015–0.016	0.95
LDL	0.022	0.0058–0.038	0.0080	0.020	0.0033–0.036	0.019	0.023	0.0062–0.039	0.0075
Homocysteine	-0.042	-0.18–0.095	0.54	-0.046	-0.19–0.094	0.52	-0.056	-0.20–0.088	0.44
RHI	-0.012	-0.024–0.00062	0.062	-0.012	-0.025–0.0010	0.070	-0.011	-0.024–0.0019	0.092
AI@75	0.47	0.22–0.72	<0.001	0.42	0.17–0.66	0.0013	0.42	0.16–0.67	0.0018
IL-8	0.22	-0.049–0.50	0.11	0.24	-0.036–0.52	0.086	0.24	-0.050–0.52	0.10
MCP-1	-0.30	-0.73–0.13	0.17	-0.28	-0.73–0.26	0.21	-0.36	-0.81–0.083	0.11
MIP-1β^c^	0.0036	-0.0037–0.011	0.33	0.0024	-0.0050–0.0097	0.53	0.0017	-0.0059–0.0092	0.66
VEGF	0.50	-0.69–1.7	0.41	0.57	-0.65–1.8	0.35	0.45	-0.80–1.7	0.48

Effect estimates presented are β-values for years working as a welder derived from general linear models for each outcome.

^a^ The partly adjusted model was adjusted for BMI.

^b^ The fully adjusted model was adjusted for BMI, family history of CVD, and current residence.

^c^ SAA and MIP-1β were natural log-transformed.

**Table 5:** The effect of years working as a welder on blood pressure and biomarkers in welders with age adjustment.

	Unadjusted			Partly adjusted^a^			Fully adjusted^b^		
Outcome	Beta	95% CI	P	Beta	95% CI	P	Beta	95% CI	P
Systolic BP	0.39	0.17–0.61	<0.001	0.48	0.15–0.82	0.0048	0.39	0.054–0.72	0.023
Diastolic BP	0.35	0.19–0.51	<0.001	0.28	0.044–0.52	0.021	0.21	-0.026–0.45	0.080
CRP	-0.0042	-0.045–0.037	0.84	-0.038	-0.099–0.024	0.23	-0.022	-0.085–0.041	0.49
SAA^c^	0.0036	-0.011–0.018	0.63	0.00036	-0.022–0.22	0.97	0.00026	-0.023–0.024	0.98
LDL	0.022	0.0058–0.038	0.0080	-0.017	-0.039–0.0055	0.14	-0.013	-0.036–0.010	0.28
Homosysteine	0.039	-0.021–0.099	0.20	0.076	-0.014–0.17	0.096	0.073	-0.022–0.17	0.13
RHI	-0.012	-0.024–0.00062	0.062	-0.010	-0.029–0.0092	0.31	-0.0083	-0.028–0.012	0.41
AI@75	0.47	0.22–0.72	<0.001	-0.18	-0.51–0.16	0.30	-0.23	-0.58–0.12	0.20
IL-8	0.22	-0.049–0.50	0.11	0.035	-0.38–0.45	0.87	-0.0040	-0.44–0.43	0.99
MCP-1	-0.30	-0.73–0.13	0.17	-0.41	-1.1–0.25	0.22	-0.62	-1.3 –-0.059	0.073
MIP-1β^c^	0.0036	-0.0037–0.011	0.33	0.00000066	-0.011–0.0011	1.0	-0.019	-0.013–0.010	0.74
VEGF	0.50	-0.69–1.7	0.41	0.76	-1.1–2.6	0.41	0.47	-1.4–2.4	0.63

Effect estimates presented are β-values for years working as a welder derived from general linear models for each outcome.

^a^ The partly adjusted model was adjusted for age and BMI.

^b^ The fully adjusted model was adjusted for age, BMI, family history of CVD, and current residence.

^c^ SAA and MIP-1β were natural log-transformed.

Exposure-response relationships were evaluated between respirable dust and the cardiovascular markers. BP was not associated with the concentration of PM. SAA was significantly positively associated with respirable dust. For the other markers, the beta estimates indicated that there were increases of CRP, LDL, AI@75, and MIP-1β, but none of these associations was significant (Table 6).

**Table 6:** The effect of respirable dust on blood pressure and biomarkers in welders.

	Unadjusted			Partly adjusted^b^			Fully adjusted^c^		
Outcome	Beta	95% CI	P	Beta	95% CI	P	Beta	95% CI	P
Systolic BP	-0.0030	-1.5–1.5	1.0	-0.25	-1.7–1.3	0.74	-0.58	-2.0–0.84	0.42
Diastolic BP	0.24	-0.89–1.4	0.67	-0.067	-1.1–1.0	0.90	-0.29	-1.3–0.74	0.58
CRP	0.16	-0.10–0.43	0.22	0.15	-0.11–0.42	0.26	0.19	-0.077–0.45	0.16
SAA^d^	0.13	0.033–0.22	0.0082	0.13	0.032–0.22	0.0090	0.13	0.032–0.22	0.0092
LDL	0.080	-0.027–0.19	0.14	0.040	-0.056–0.14	0.41	0.052	-0.045–0.15	0.29
Homocysteine	-0.067	-0.95–0.82	0.88	0.046	-0.85–0.94	0.92	0.0072	-0.91–0.92	0.99
RHI	-0.030	-0.11–0.052	0.47	-0.019	-0.10–0.064	0.65	-0.015	-0.099–0.069	0.72
AI@75	1.2	-0.48–2.9	0.16	0.48	-0.97–1.9	0.52	0.38	-1.1–1.9	0.61
IL-8	-0.54	-2.3–1.3	0.55	-0.85	-2.6–0.94	0.35	-0.94	-2.8–0.89	0.31
MCP-1	-0.39	-3.2–2.4	0.78	-0.27	-3.1–2.6	0.85	-0.70	-3.5–2.2	0.63
MIP-1β^d^	0.039	-0.071–0.086	0.096	0.036	-0.011–0.082	0.13	0.032	-0.015–0.080	0.18
VEGF	-3.4	-11–4.4	0.39	-3.8	-12–4.1	0.35	-4.4	-12–3.6	0.27

Effect estimates presented are β-values for respirable dust derived from general linear models for each outcome.^a^

^a^ Use of mask was taken into account in the estimation of the exposure to respirable dust.

^b^ The partly adjusted model was adjusted for age and BMI.

^c^ The fully adjusted model was adjusted for age, BMI, family history of CVD, and current residence.

^d^ SAA and MIP-1β were natural log-transformed.

## Discussion

This study showed that welders had increased systolic and diastolic BPs compared with controls, and their BP increased significantly with years working as a welder. Also, IL-8, a marker for neutrophil activation was elevated in the welders. The welders were exposed to 10-fold higher levels of PM compared with controls. However, the concentrations of PM for most of the welders (97 out of 101) were still below the Swedish occupational exposure limit (5 mg/m^3^) [41]. Our findings therefore suggest that workers with low-to-moderate exposure to welding fumes still have higher risk of CVD, despite precautionary measures.

This study had multiple strengths. Only non-smokers were included and therefore, the confounding effect of current smoking was, to a large extent, removed. This study was restricted to male welders because we had limited opportunities to obtain a group of female welders large enough to evaluate sex-specific effects of welding fumes on CVD risk. However, since it has been suggested that women could be more susceptible to PM-induced CVD risk [42, 43], our results showing increased BP in male welders might indicate that female welders could face an even higher risk of CVD. The different companies used similar techniques for welding, leading to homogenous composition of welding fumes. By using portable equipment, we were able to measure endothelial function in the field in a non-invasive way; to our knowledge, this is the first study to measure endothelial function in the field in an occupational setting.

This study also had some limitations. The study was cross-sectional, but information regarding various characteristics of the participants was gathered in order to take possible confounders into account. Measurements of both exposure and markers of effect were performed only once. Therefore, the estimate of exposure to respirable dust was rather uncertain. The measurement of BP was performed once on each participant. However, all participants rested for 15 minutes before the measurement of BP, so we do not expect large errors in the measurement of BP. Other biomarkers were also measured only once. Therefore the results might be affected by intra-individual variation.

The study participants had an average systolic BP in the “high normal” range according to ESH-ESC Guidelines [40], probably because many workers were overweight. Still, the welders showed higher BP (median = 130 mm Hg for systolic and 75 mm Hg for diastolic BP) compared to controls (median = 125 mm Hg for systolic and 70 mm Hg for diastolic BP). The increased BP might be due to difference in physical workload; however, since the welders and controls rested well before the measurement, this was not likely. Since the prevalence of CVD history in welders and controls did not differ, and the BP differences were still significant in the sensitivity analysis excluding participants with CVD history, one could infer that the difference of BP between welders and controls was not due to previous incidence of CVD. Furthermore, heart rate was also compared, and no significant difference in heart rate between welders and controls was found. This indicated that the increased BP in welders was not due to differences in physical stress.

Persons with BP in the range of 120-139/80-89 mm Hg are more likely to develop hypertension later, and studies as early as 1939 reported that they had about twice the risk of mortality compared to persons with lower blood pressure [44]. The risk increases along with increased BP; persons with BP 130-139/85-89 mm Hg had threefold higher risk of developing hypertension and twofold increased risk of CVD independent of progression to hypertension, while persons with BP 120-129/80-84 mm Hg also had excessive risk, but to a lesser extent [45]. Our results indicated that our study participants were at risk of CVD, and the welders had an even higher risk.

We identified a significant association between elevated BP and years working as a welder, but found no association with the concentration of welding fumes. An acute increase in BP has been associated with short-term PM exposure in several studies [46, 47]; the overall estimation was that a 0.01 mg/m^3^ increase in fine particles can raise BP by 1–5 mm Hg [47]. We did not observe such an association in our study. This could be because the workers’ exposure to PM was chronic rather than short-term. The results from this study are difficult to compare to the results from the literature, since there is little information about the association between long-term exposure to welding fumes and its effect on BP. In the sensitivity analysis where participants with CVD history were excluded, we still observed a significant association between diastolic BP and working years as a welder, although the association between systolic BP and working years as a welder became non-significant. This might be due to the reduced sample size.

A few studies have investigated the inflammatory response induced by welding fumes with inconsistent results. Järvelä et al. found a slight, acute inflammatory effect indicated by an increase of leukocytes and neutrophils in blood and a decrease of IL-1β, which were measured before and after work shifts in 20 workers [48]. They did not observe changes of concentrations of CRP, IL-6, IL-8, or TNF-α. Scharrer et al. reported a significant decrease of endothelin-1 after 1 hour of exposure to welding fumes of 3.5 mg/m^3^ in 20 non-smoking, healthy volunteers, but they observed no changes in leukocyte count, CRP, TNF-α, IL-6, or IL-8 [49]. Kim et al. found increased CRP 16 hours after exposure to welding fumes in 37 workers [50]. Long-term exposure to mild steel welding has also been associated with local neutrophil inflammation of the lungs, as well as an increased expression of the gene encoding VEGF, and decreased expression of the gene encoding hemeoxygenase-1, which functions in a pathway involved in oxidative stress [51]. Recently, an association between welding fume nanoparticles and the pro-inflammatory secretome was shown in human macrophages, but not human primary lung fibroblasts [52]. These studies, together with ours, show limited evidence of an influential inflammatory response induced by exposure to welding fumes.

No difference of endothelial function or arterial stiffness was observed in this study. The association between endothelial function and particle exposure has been investigated by various studies; however, the results were inconsistent. Forchhammer et al. found no change of endothelial function by wood smoke exposure [53]; Pope et al. did not find an effect on endothelial function of short-term exposure to wood or coal smoke, but of the last 48 hours of ambient particle levels [54]; Bonetti et al. found an effect of secondhand smoke on endothelial function [55]; Allen et al. saw an effect on endothelial function of filtering the air in the homes of wood burners [56]; Bräuner et al. saw an effect of filtering air in homes in Copenhagen among the elderly but not the young [18, 57]. Arterial stiffness has also been reported to increase after exposure to particles [58–60]. However, all these reports were based on short-term exposure, which makes direct comparisons difficult.

## Conclusion

In conclusion, we found that long-term low-to-moderate exposure to welding fumes was associated with higher blood pressure. This finding highlights that further precautionary measures need to be taken for occupational exposure to particles.
